# Fluorescent Ratiometric Indicators Based on Cu(II)-Induced Changes in Poly(NIPAM) Microparticle Volume

**DOI:** 10.3390/s130101341

**Published:** 2013-01-21

**Authors:** John Osambo, W. Rudolf Seitz, Daniel P. Kennedy, Roy P. Planalp, Aaron M. Jones, Randy K. Jackson, Shawn Burdette

**Affiliations:** 1 Department of Chemistry, University of New Hampshire, Durham, NH 03824, USA; E-Mails: john.osambo@colby-sawyer.edu (J.O.); dpkennedy79@gmail.com (D.P.K.); roy.planalp@unh.edu (R.P.P.); 2 Department of Chemistry, University of Connecticut, Storrs, CT 06269, USA; E-Mails: amark321@gmail.com (A.M.J.); randy.jackson@uconn.edu (R.K.J.); 3 Department of Chemistry and Biochemistry, Worcester Polytechnic Institute, Worcester, MA 01609, USA; E-Mail: scburdette@wpi.edu

**Keywords:** fluorescence resonance energy transfer, copper, polyNIPAM, microparticles, ratiometric indicator, metal complex

## Abstract

Microparticles consisting of the thermal responsive polymer *N-*isopropyl acrylamide (polyNIPAM), a metal ion-binding ligand and a fluorophore pair that undergoes fluorescence resonance energy transfer (FRET) have been prepared and characterized. Upon the addition of Cu(II), the microparticles swell or contract depending on whether charge is introduced or neutralized on the polymer backbone. The variation in microparticle morphology is translated into changes in emission of each fluorophore in the FRET pair. By measuring the emission intensity ratio between the FRET pair upon Cu(II) addition, the concentration of metal ion in solution can be quantified. This ratiometric fluorescent indicator is the newest technique in an ongoing effort to use emission spectroscopy to monitor Cu(II) thermodynamic activity in environmental water samples.

## 1. Introduction

The effects of metal ions in biological and environmental systems depend on thermodynamic activity rather than total concentration [1]. For example, Cu(II) toxicity varies over an order of magnitude depending on its degree of complexation with organic matter and competition with other metal ions for binding sites on aquatic organisms [2,3]. The biotic ligand model corrects for these effects and predicts Cu(II) toxicity better than total copper measurements [2,3].

While few techniques measure the thermodynamic activity of a metal ion directly, indicators provide a convenient method to quantify this property. Metal ion binding to a ligand (receptor) in an indicator induces measurable changes in the optical properties of a reporting group that may or may not be connected to the ligand. To prevent perturbing the metal ion activity, indicator concentration must remain lower than the total metal ion concentration. The measurable range of analyte concentration for any indicator depends on the receptor's affinity for the metal ion of interest, and is defined as log K_f_ − 1 to log K_f_ +1, where K_f_ is the conditional formation constant for metal ion.

The high sensitivity of emission spectroscopy means indicators utilizing fluorescence can be employed at the lowest possible levels. Furthermore, ratiometric fluorescence indicators simplify signal calibration by monitoring changes at different emission wavelengths rather than the absolute intensity. By measuring the intensity ratio at two different wavelengths, the output remains independent of indicator concentration. Ratiometric indicators are essential for applications such as measuring intracellular metal ion activity where total indicator concentrations cannot be established accurately. Ratiometric fluorescent indicators have enabled researchers to study the biological role of Ca(II) [4] and Zn(II) [5]; however, designing ratiometric indicators for metal ions such as Cu(II) that quench fluorescence emission remains challenging.

We and others are developing fluorescent metal ion indicators based on the thermal phase transition of poly(*N-*isopropylacrylamide) (polyNIPAM) [6–11]. PolyNIPAM undergoes a thermal phase transition at elevated temperatures, which leads to aggregation and precipitation [12]. The temperature at which the phase transition occurs is defined as the lower critical solution temperature (LCST). Metal ion binding to a polymer-bound ligand can modulate LCSTs by either introducing or neutralizing charge on the macromolecular backbone. With a properly engineered polymer maintained at a specific temperature, metal ion binding can induce the polyNIPAM thermal phase transition. When fluorophores are included in the polyNIPAM formulation, the phase transition can be coupled to emission changes. An important advantage of this approach is that selectivity depends on the relative affinity of the ligand for different metal ions [9], which implies that selectivity can be varied by using different ligands.

Ratiometric indicators can be designed using an environmentally sensitive fluorescent label [7,8,10]. In order to get a ratiometric response, we employed a fluorophore that shifts emission wavelength as function of polarity; however, the indicator was sensitive to slight changes in temperature and required UV excitation [6]. Alternatively, fluorescence resonance energy transfer (FRET) between pairs of fluorophores with specific photophysical properties can be employed to obtain a ratiometric response. Recently, we successfully coupled the thermal phase transition of polyNIPAM with FRET signaling to measure Cu(II) concentrations with an uncrosslinked polymer indicator, which is soluble below the LCST [9]. While this study provides important proof-of-concept detection, a homogenous indicator cannot be reused and is not useful for continuous sensing.

In contrast to solution-based indicators, detection systems using a solid support such as microparticles can be recycled and readily integrated into continuous sensing devices [13,14]. Lightly cross-linked polyNIPAM microparticles containing dibenzo-18-crown-6 ligands labeled with a FRET pair responds to K^+^[11]. The charge introduced by K^+^ binding causes microparticles to swell, which increases the average distance between the donor and acceptor fluorophores of the FRET pair. The emission ratio of acceptor to donor intensities can be used to calculate potassium ion activity. While this study represents a significant advance in indicator design, K^+^ does not quench emission like Cu(II). Homogeneous polyNIPAM indicators can overcome the inherent quenching properties of Cu(II) [6,9]; however, there is no experimental evidence to suggest the same effects are possible in a microparticle where molecular motion is more restricted. In addition to photophysical considerations, the K^+^ indicator only examines introduction of charge onto the polymer backbone and does not address a charge neutralization mechanism, which would cause microparticles to collapse.

## 2. Experimental Section

### 2.1. Ligand Synthesis

#### 2.1.1. Materials and Methods

Dichloromethane was sparged with argon and dried by passage through a Seca Solvent Purification System. The synthesis of *N*-(4′-methyl-[2,2′]bipyridinyl-4-ylmethyl)-*N*-propyl-acrylamide (**1**) was performed as previously described [9]. All other materials were purchased and used as received. All reactions were carried out under a dry nitrogen atmosphere. Flash chromatography was performed with silica gel-60 (230–400 mesh). Thin layer chromatography (TLC) analysis was performed with Silicycle F254 silica gel-60 plates or Merck F254 aluminum oxide-60 plates and viewed with UV light or developed with ninhydrin stain. CDCl_3_ was obtained from Cambridge Isotope Laboratories. ^1^H and ^13^C NMR spectra were recorded on a Bruker DRX400 (Karlsruhe, Germany) or Varian Mercury 400 MHz NMR (Palo Alto, CA, USA) at ambient probe temperature, 283 K, and referenced to residual solvent peaks or internal TMS. The NMR spectra are expressed on the *δ* scale. Proton chemical shifts are annotated as follows: ppm (integral, multiplicity, coupling constant in Hz). IR spectra were recorded on a Nicolet 205 FT-IR (Madison, WI, USA) instrument as KBr pellets or thin films on KBr plates. High Resolution mass spectra were carried out at the University of Notre Dame Mass Spectrometry Facility.

#### 2.1.2. [*Tert*-butoxycarbonyl methyl-(3-vinyl-phenyl)-amino] acetic acid tert-butyl ester (**2**)

To CH_3_CN (15.0 mL) was added 3-vinylaniline (476 mL, 4.20 mmol), proton sponge (1.98 g, 9.24 mmol), and NaI (0.316 g, 2.11 mmol) sequentially. The resulting mixture was stirred magnetically for 5 min at room temperature affording a colorless solution. To the solution was then added *tert*-butyl bromoacetate (1.30 mL, 8.82 mmol) resulting in the immediate formation of a white precipitate. The mixture was refluxed for 51 h. The heterogeneous mixture was cooled, poured into 100 mL of EtOAc, and filtered through a glass frit. The filtrate was collected and washed with an equal volume of brine. The organic layer was isolated, dried over excess MgSO_4_, filtered via gravity through paper, and the solvent removed under vacuum to provide a yellow oil. Flash chromatography on silica (2:1 hexanes–EtOAc) yielded colorless oil (1.24 g, 85.0%). TLC R_f_ = 0.6 (2:1 hexanes-EtOAc). ^1^H-NMR (CDCl_3_) d 7.16 (1 H, m), 6.83 (1 H, m), 6.65 (1 H, dd, *J* = 11.0 Hz), 6.61 (1 H, m), 6.51 (1 H, m), 5.67 (1 H, dd, *J* = 0.8, 17.6 Hz); 5.19 (1 H, dd, *J* = 0.8, 11.0 Hz), 4.02 (4 H, s), 1.46 (18 H, s). ^13^C NMR (CDCl_3_) d 170.39, 148.52, 138.56, 137.68, 129.43, 116.40, 113.54, 112.30, 110.47, 81.84, 54.86, 28.26. IR (thin film): 3084.6, 2,977.7, 1,741.7, 1,599.6, 1,151.2 cm^−1^. HRMS (ESI): Calcd for MH^+^, 347.2097; Found 347.2117.

### 2.2. Polymer Synthesis

#### 2.2.1. Materials and Methods

*N*-Isopropylacrylamide (NIPA), *N,N′*-methylene-bis-acrylamide, 2,2′-azoisobutyrylnitrile (AIBN) and acetonitrile (CH_3_CN) were purchased from Aldrich Chemical Company (Milwaukee, WI, USA); poly(styrene-co-acrylonitrile) was purchased from Scientific Polymer Product Inc (Ontario, NY, USA); and 2-naphthylmethacrylate (NMA) and 9-vinylanthracene (VAN) were purchased from Polyscience Inc. (Warrington, PA, USA). All reagents were used as received.

#### 2.2.2. Poly(*N-*isopropylacrylamide-co-*N-*((4-methyl-2,2-bipyridine-4-yl)methyl)-*N*-propyl-acrylamide) Labeled with NMA and VAN (**3**)

*N*-Isopropylacrylamide (1.018 g, 9 mmol), **1** (148 mg, 0.5 mmol), *N*,*N′*-methylene-bis-acrylamide (77.1 mg, 0.5 mmol), poly(styrene-co-acrylonitrile) (224 mg, avg. MW 185,000), NMA (2.12 mg, 0.01 mmol), VAN (205 μg, 1 μmol) and AIBN (0.33 g, 0.20 mmol) were dissolved in CH_3_CN (50 mL), and the resulting mixture was sonicated for 30 min to effect solvation. After purging the reaction vessel with N_2_, the reaction mixture was stirred at 60 °C for 16 h. The resulting polymer particles were separated by centrifugation, re-suspended three times in CH_3_CN to remove unreacted monomer, and washed with water several times to remove CH_3_CN.

#### 2.2.3. Poly(*N*-isopropylacrylamide-co-tert-butoxycarbonylmethyl-(3-vinyl-phenyl)amino)-acetic acid *tert*-butyl ester) Labeled with NMA and VAN (**4**)

The synthesis of **4** followed analogous procedures to those used for **3** except 0.5 mmol of **2** was used in the formulation instead of **1**.

#### 2.2.4. Poly(*N-*isopropylacrylamide-co-carboxymethyl-(3-vinyl-phenyl)amino)-acetic acid) Labeled with NMA and VAN (**5**)

The *t*-butyl esters of **4** were removed by suspending the polymer particles in 1 M H_2_SO_4_, and heating the mixture to 40–45 °C for 8 h. The polymer particles were isolated by centrifugation. The hydrolysis process was repeated to ensure complete hydrolysis of the esters. After hydrolysis, the particles were suspended in deionized water, stirred for 30 min at room temperature and centrifuged to isolate the polymer. This isolation process was repeated five times and the pH of filtrate (water) was tested after each iteration of washing.

### 2.3. Spectroscopic Methods

#### 2.3.1. Microparticle Morphology

Microparticle morphology was determined using an Amray 3300FE scanning electron microscope (Bedford, MA, USA).

#### 2.3.2. Fluorescence Titration of **3** with Cu(II)

A known mass of dry polymer particles was suspended in a known volume of 0.1 M MOPS buffer at pH 6 and stirred vigorously for 4–6 h. A 2.5 mL portion of the polymer suspension was transferred into a quartz cell and inserted into a Cary Eclipse spectrofluorometer. The suspended particles were titrated with 10^−5^ M standardized Cu(II) at both 25 °C and 50 °C. The titration procedure was performed by adding 20 μL aliquots of standard Cu(II) solution into the polymer suspension. The intensities of the two fluorophores were obtained by exciting the particles at 260 nm. The fluorescence intensity ratio of VAN:NMA was calculated after addition of each aliquot and a graph of fluorescence intensity ratio of VAN:NMA was plotted against the Cu(II) concentration for the two temperatures. Measurements were performed at pH 6 to prevent ligand protonation and avoid the formation Cu(II) hydroxide complexes. At lower pH, ligand protonation will compete with metal ion binding and reduce the ligand affinity for Cu(II). Ligand protonation also affects the degree of microparticle swelling in the absence of Cu(II). If the pH is increased, Cu(II) will form hydroxide complexes, reducing the free Cu(II) concentration).

#### 2.3.3. Fluorescence Titration of **5** with Cu(II)

An analogous procedure to that used for the titration of **3** was used, with 10^−3^ M Cu(II).

#### 2.3.4. Potentiometric Titration of **3** with Cu(II) and Determination of Formation Constants (K_f_)

A known mass of **3** particles were suspended in known volume of MOPS buffer at pH 6 with ionic strength adjusted to 0.1 M using KCl. The polymer suspension (50 mL) was titrated potentiomentrically with 10^−5^ M Cu(II) using a Cu(II)-selective electrode.

#### 2.3.5. Potentiometric Titration of **5** with Cu(II) and Determination of Formation Constants (K_f_)

A titration process analogous to that performed with **3** was used except with 10^−3^ M Cu(II).

## 3. Results and Discussion

In earlier studies, the thermal phase transition properties of polyNIPAM were applied successfully to the design of homogeneous ratiometric indicators for Cu(II) [6,9]. These indicators exploit temperature-induced polymer architecture changes in the signal transduction mechanism. Below the LCST of 32 °C, pure polyNIPAM remains soluble in water because of hydrogen bonding between water and the amide groups; however, the polymer collapses and precipitates from solution at higher temperatures when the hydrogen bonds are broken [15]. By including a Cu(II)-binding ligand and a fluorophore pair capable of undergoing FRET in the polymer formulation, the aggregation/disaggregation can be coupled to fluorescence ratio changes by shifting the LCST.

FRET involves distance-dependent transfer of excitation energy between a donor and acceptor fluorophore. When the donor and acceptor are in close proximity, excitation of the donor fluorophore leads to fluorescence characteristic of the acceptor. Alternatively, excitation of the donor fluorophore leads to donor-based emission if the FRET pair is separated in space. The ratio of donor emission to acceptor fluorescence correlates with the distance between the fluorophores, which depends on temperature in polyNIPAM. When Cu(II) binds to neutral ligands on the polyNIPAM, the additional charge-based hydrophilic interactions shift the LCST to higher temperatures; therefore, the decreased ratio of acceptor/donor emission correlates directly with Cu(II) concentration. Alternatively, a Cu(II)-binding negatively charged ligand will shift the LSCT to lower temperatures as the overall charge on the polymer backbone is neutralized making the macromolecule more hydrophobic, and the acceptor/donor emission ration increases.

**Figure 1.**
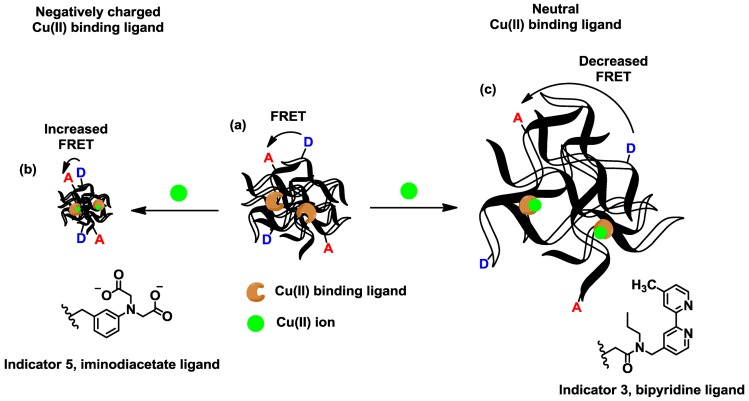
Signaling mechanism in polyNIPAM particle indicators. (**a**) Cross-linked polyNIPAM particles are functionalized with a Cu(II) binding ligand and a fluorophore pair capable of undergoing FRET. (**b**) If the Cu(II) binding ligand carries a negative charge, metal ion binding neutralizes the charge causing the particles to shrink, which decreases the distance between the donor and acceptor fluorophores and increases FRET. (**c**) When a neutral Cu(II) binding ligand is incorporated into the particles, the introduction of charge on the polymer backbone causes the particles to swell. The increased distance between the donor and acceptor fluorophores leads to decreases in FRET.

Cross-linked polyNIPAM microparticles swell in water below the LCST and contract at higher temperatures (Figure 1). As with uncrosslinked polyNIPAM, including comonomers in the formulation shifts the LCST of the polyNIPAM particles to lower or higher values depending on hydrophilic/hydrophobic interactions. A small percentage of a negatively charged monomer, like a carboxylate unit, can completely eliminate the phase transition resulting in microparticles that are swollen at all temperatures. Cu(II) binding to the ligands on the polymer backbone shifts the LCST by modifying the number of charges on the polymer backbone and induces swelling or shrinking. The temperature-dependent morphology changes cause the average distance between donor and acceptor fluorophores to increase or decrease, which changes the extent of FRET and therefore the emission ratio.

**Figure 2.**
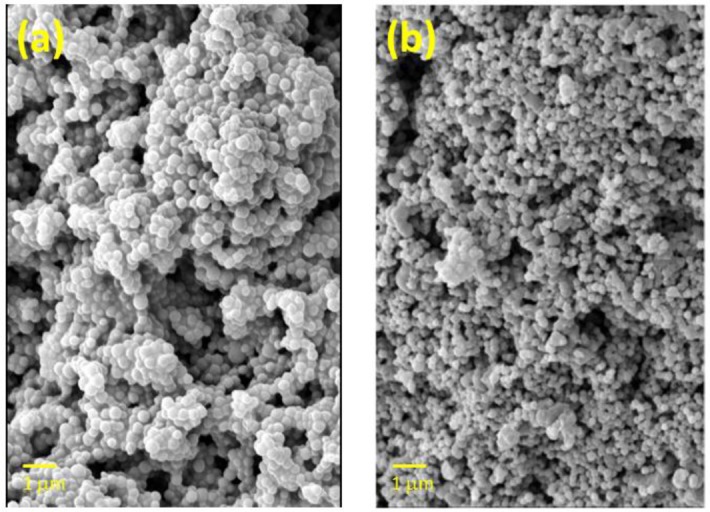
SEM images of polyNIPAM polymer indicators. (**a**) Indicator **3** containing neutral bipyridine ligand. (**b**) Indicator **4** containing iminodiactate esters.

**Scheme 1.**
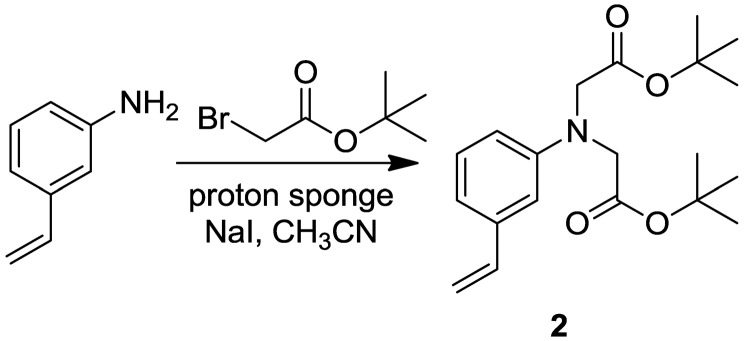
Synthesis of **2**.

Copolymer microparticles were prepared by dispersion polymerization at 60 °C in CH_3_CN using AIBN as the initiator and a styrene/acrylonitrile copolymer as a stabilizer. Indicator particles were obtained from polymeric formulations containing ca. 5 mol% ligand (**1** or **2**), 5 mol% methylene-bisacrylamide cross-linker, 90 mol% NIPA, 0.2 mol% naphthalene methacrylate and 0.05 mol% VAN (Figure 2). The microparticle diameter for indicator 1 ranges from 0.28 to 0.35 μm with an average diameter of 0.32 μm. Most indicator **2** microparticles have a diameter close to 0.15 μm but there is a subpopulation of larger microparticles. Bipyridine and phenyliminodiacetate were selected as the Cu(II)-binding ligands because of their well-studied coordination chemistry as well as their contrast in charge at neutral pH. The bipyridyl acrylate **1** was prepared as previously described [9], and copolymerized with NIPA and fluorophores using the above formulation to make indicator **3**. The phenyliminodiacetate was prepared as the *t*-butyl ester **2** (Scheme 1) and copolymerized with NIPA by the vinyl group. Treatment of the ester-containing polymer with acid provided the iminodiacetate containing indicator **5**.

NMA and VAN were included in the polymer formulation as the FRET pair [16], and were chosen because both monomers are commercially available and because xanthene-based fluorophores like rhodamine and fluorescein initiate radical polymerizations, which could lead to unwanted radical transfer and heterogeneity in microparticles [17,18]. To avoid complications in preparing the first generation indicators, the NMA and VAN fluorophore, which are compatible with free radical conditions, were ideal. Since the vinyl group of VAN reacts more rapidly than the acrylate of NMA, the VAN fluorophore will tend to localize near the center of the microparticles while the NMA will be distributed more evenly through the polymer. Fluorescence measurements involved exciting NMA at 260 nm, and measuring the ratio of VAN emission at 421 nm to NMA emission at 355 nm. While this FRET pair is convenient, the short excitation and emission wavelengths are subject to interference in many types of samples. Future efforts will be focused on labeling microparticles post-polymerization with fluorophores having excitation and emission in the visible region of the spectrum.

In the absence of a charged group, the emission ratio of FRET pair labeled polyNIPAM microparticles changes as a function to temperature. With excitation at 260 nm, emission from both NMA at 355 nm and VAN at 421 nm was observed. Since the absorption band of VAN minimally overlaps with the 260 nm excitation light, a significant fraction of the VAN emission arises from FRET from NMA. The emission intensity of both NMA and VAN decrease with increasing temperature in part because of thermal quenching. Some portion of the emission decrease may be due to particle settling that reduces the amount of polymer in the optical path; however, control experiments measuring second order scattered radiation suggest that settling occurs on the hour time scale and is not accompanied by a change in intensity ratio. The addition of Cu(II) at constant temperature caused a change in intensity ratio, but did not affect absolute intensities significantly, indicating little, if any, Cu(II) quenching.

**Figure 3.**
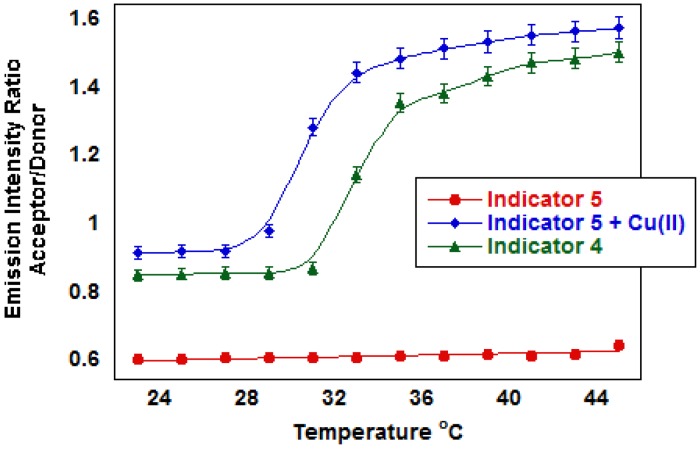
Changes in emission ratio due to FRET in a charged carboxylate ligand (**5**) *vs.* an uncharged ester (**4**) polyNIPAM indicator as a function of temperature. Cu(II) binding neutralizes charge on the polymer backbone of **5**, which contain negatively charged carboxylate groups, and causes emission ratio changes that closely resemble that of **5**, which contains neutral ester groups.

While both the NMA and VAN emission intensities decrease, NMA decreases more significantly. As temperature increases, NMA excitation energy transfers to VAN through FRET (Figure 3). The ratio changes from 0.92 at 25 °C to 1.6 at 35 °C. The LCST, estimated as the midway point of the change in ratio, occurs at 30 °C. The indicator LSCT is ∼2 °C below the LCST for unmodified polyNIPAM, which shows the impact of VAN, NMA and other hydrophobic monomers. The emission intensity ratio also changes over a wider range of temperature than unmodified polyNIPAM, which suggests the cross-linker introduces polymer heterogeneities that broaden the thermal phase transition. For comparison, the emission ratio remains unchanged over the same temperature range for indicator **5**, which contains negatively charged groups. When Cu(II) neutralizes the charge carried on indicator **5**, the emission ratio changes are similar to the uncharged polymer.

**Figure 4.**
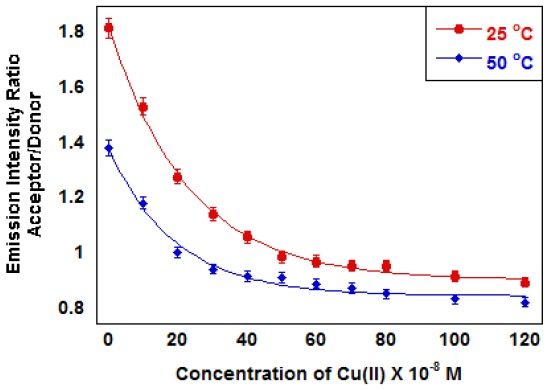
Emission Intensity ratio *vs.* [Cu(II)] for indicator **3** at 25 and 50 °C (MOPS, pH 6.0). The decrease in emission ratio is consistent with reduced FRET as particles swell when charge is introduced on the polymer backbone by bound Cu(II).

In MOPS buffer at pH 6 indicator **3** exhibits intensity ratio changes characteristic of FRET upon the addition of Cu(II) (Figure 4). Fluorescence experiments were carried out at pH 6.0 to prevent the formation of metal hydroxide complexes. The intensity ratio decreases with added Cu(II) up to ∼5 × 10^−5^ M. Above this concentration, the intensity ratio remains constant. The measured endpoint correlates closely with a 1:1 complex based on the calculated ligand concentration in the microparticles. The intensity ratio changes at both 25 °C and 50 °C, but more significantly at 50 °C. The decrease in acceptor emission, which indicates a decrease in FRET, is consistent with the microparticles swelling due to the introduction of charge by Cu(II) binding to the polymer backbone.

**Figure 5.**
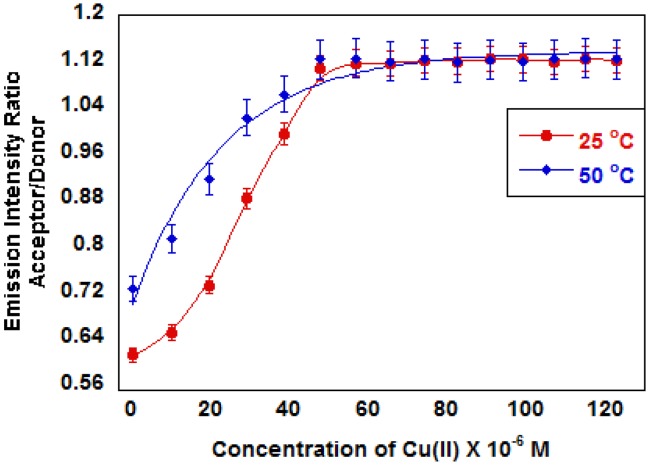
Emission Intensity ratio *vs.* [Cu(II)] for indicator **5** at 25 and 50 °C (MOPS, pH 6.0). The increase in emission ratio is consistent with reduced FRET as particles contract as the ligand charge is neutralized on the polymer backbone by Cu(II) binding.

In indicator **5** containing the negatively charged iminodiacetate ligand, the emission intensity ratio increases as Cu(II) concentrations increase at both 25 °C and 50 °C (Figure 5). At pH 6.0 where both carboxyl groups are deprotonated, the polyNIPAM microparticles swell due to the charges on the polymer, and no thermal phase transition was observed up to 50 °C. Addition of Cu(II) restores the thermal phase transition as charge neutralization causes the particles to contract increasing FRET. Indicator **4** where the carboxylate groups are protected by ester groups exhibits thermal phase transition behavior similar to the microparticles containing no ligand (LCST ∼30 °C). The Cu(II) complex of indicator **5** exhibits less hydrophobicity than indicator **4** as illustrated by the 33 °C LCST.

When indicator **3** was titrated with Cu(II) using an ion selective electrode, the titration endpoint suggests a bipyridine ligand content of 84% of the nominal concentration calculated from the initial formulation. The formation constant was determined to be 2.6 × 10^7^ M, consistent with the literature value of 1.0 × 10^8^ for the 1:1 complex of Cu(II) and bipyridine. The working range of any metal ion indicator is pM = log K_f_ ± 1, so the microparticles can detect Cu(II) activities between 4 × 10^−9^ and 4 × 10^−7^ M. Outside this range the change in intensity ratio will be too small to detect with adequate precision. Experiments with the ion selective electrode and indicator **5** indicate a ligand content of 88% based on initial formulation, and an 8.4 × 10^5^ M formation constant for a 1:1 complex, which is close to the theoretical value of 2.5 × 10^6^. This indicator will measure Cu activities from 1.2 × 10^−7^ to 1.2 × 10^−5^ M. The affinity data is consistent with the values obtained for the corresponding homogeneous, linear indicators [9] so the microparticle systems should have similar selectivity for Cu(II), which is independent of the macromolecule and based primarily on the Irving-Williams series. For this proof-of-concept system, the lack of readily available ion selective electrodes precludes proving absolute formation constants for every possible competing metal ion. Our studies suggest that when the optimal Cu(II)-selective ligand becomes available, integrating it into either a homogeneous linear polymer or a microparticle will provide an indicator suitable for the desired applications [6,9].

The data in Figures 4 and 5 include measurements above and below the polyNIPAM LCST. The fluorescence ratio change is larger below the LCST, which suggests that the responses are based on charge-induced polymer swelling. Detection limits for a given indicator will depend on the metal ion-indicator formation constant and our ability to measure fluorescence from a microparticle suspension. The data in Figure 4 show a measurable ratio change for less than 1 μM metal ion using indicator **3**. The data in Figure 5 show a measurable ratio change for concentrations on the order of 1 μM.

## 4. Conclusions

We have prepared lightly cross-linked derivatized microparticles that contain a Cu(II)-binding ligand and pair of fluorophores that undergo FRET. Using the neutral ligand bipyridine, metal ion binding introduces a charge on the microparticle that causes swelling. The increase in the average distance between donor and acceptor fluorophores reduces FRET. With the charged ligand iminodiacetate, metal ion binding neutralizes the charge, causing microparticles to contract. The decrease in average distance between donor and acceptor fluorophores increases FRET. The linear relationship between the emission intensity ratio changes correlates with the fraction of ligands bound to Cu(II) in both systems. The indicator remains emissive even though Cu(II) typically quenches fluorescence, so this approach provides a significant opportunity to develop ratiometric fluorescent indicators for metal ions that have so far eluded detection using this type of approach.

In contrast to the homogeneous intermolecular indicator approach [9], this solid phase system involves responds at room temperature and demonstrates a response using a ligand charge neutralization in addition to charge introduction upon Cu(II) binding to the ligand. While the current system utilizes fluorophores requiring short wavelength excitation, future efforts will address methodologies to incorporate alternative FRET pairs into next generation indicators.
